# Elbasvir/Grazoprevir: A Review of the Latest Agent in the Fight against Hepatitis C

**DOI:** 10.1155/2016/3852126

**Published:** 2016-06-15

**Authors:** Allison M. Bell, Jamie L. Wagner, Katie E. Barber, Kayla R. Stover

**Affiliations:** ^1^Department of Pharmacy Practice, University of Mississippi School of Pharmacy, Jackson, MS 39216, USA; ^2^Department of Medicine-Infectious Diseases, University of Mississippi Medical Center, Jackson, MS 39216, USA

## Abstract

Hepatitis C virus (HCV) is estimated to affect up to 150 million people worldwide. Despite worldwide prevalence, treatment modalities prior to 2011 remained suboptimal, with low virologic response rates and intolerable side effect profiles. Fortunately, the landscape of treatment for chronic hepatitis C has rapidly evolved since the introduction of HCV NS3/4 protease inhibitors in 2011. Elbasvir, a NS5A inhibitor, combined with grazoprevir, a NS3/4A protease inhibitor, is the latest FDA-approved therapy for patients with genotype 1 or 4 chronic hepatitis C, with or without compensated cirrhosis. This review will focus on the current literature and clinical evidence supporting elbasvir/grazoprevir as first-line therapy in patients with genotypes 1 and 4 chronic hepatitis C.

## 1. Introduction

Hepatitis C virus (HCV) is a single-stranded RNA virus that is estimated to affect 130–150 million people worldwide [1]. Although originally called simply “non-A, non-B hepatitis”, HCV was soon named and identified as a predominantly parenterally transmitted member of the family of flaviviruses [2].

Since it was first isolated in 1989, advances in the understanding of the HCV life cycle have led to significant improvements in the knowledge and treatment of this disease [3, 4]. Prior to 2000, the primary management of HCV included interferon monotherapy, which boasted a sustained virologic response (SVR) of 15% after 48 weeks of therapy [5]. Shortly thereafter, combination therapy with alpha 2b interferon and ribavirin boasted an SVR of 40–50%, depending on treatment experience, after 24 weeks of therapy [5]. Although results were promising compared to monotherapy, prolonged treatment durations and severe side effects limited tolerability of these medications. With the potential for shorter required treatment durations, therapy recommendations changed to triple-drug regimens in 2011 when the first direct-acting antiviral (DAA) protease inhibitors, boceprevir and telaprevir, were approved [4, 6]. In 2014, however, treatment regimens radically changed with the development of additional, more effective DAAs [4]. These agents, including sofosbuvir, simeprevir, daclatasvir, ledipasvir, and paritaprevir/ritonavir + ombitasvir + dasabuvir, target individual portions of the viral proteins, including NS3/4A, NS5B, and NS5A [4]. With significant improvements in both efficacy and safety, these agents enable interferon-free regimens with significant hope and promise for the future of this now-curable disease [7, 8]. This paper reviews the literature available for elbasvir/grazoprevir, the newest DAA option for the treatment of HCV.

## 2. Elbasvir/Grazoprevir

Elbasvir/grazoprevir (Zepatier®) is a combination product with an FDA-approved indication for the treatment of chronic HCV genotypes (GTs) 1 and 4 in adults [9]. Elbasvir is an NS5A inhibitor, preventing hepatitis C viral RNA replication and virion assembly. Median EC_50_ values range from 0.2 to 3600 pmol/L, based on genotype [10]. Grazoprevir is a protease inhibitor of HCV NS3/4A that prevents cleavage of the polyprotein necessary for replication [9, 10]. Median EC_50_ values range from 0.16 to 0.8 pmol/L [10].

### 2.1. Resistance

Virologic failure with elbasvir/grazoprevir has been seen both* in vitro *and* in vivo*.* In vitro*, elbasvir has been associated with reduced activity in the presence of NS5A substitutions M28A/G/T, Q30D/E/H/K/R, L31M/V, H58D, and Y93C/H/N in genotype 1a, L28M, L31F, and Y93H in GT 1b, and L30S, M31V, and Y93H in GT 4 [9]. Grazoprevir has been associated with reduced activity in the presence of NS3 substitutions Y56H, R155K, A156G/T/V, and D168A/E/G/N/S/V/Y in GT 1a, F43S, Y56F, V107I, A156S/T/V, and D168A/G/V in GT 1b, and D168 A/V in GT 4 [9].

In phases 2 and 3 of clinical trials, treatment-emergent virologic failures, associated with amino acid substitutions, were identified in 37 patients with GT 1a, 8 patients with GT 1b, and 5 patients with GT 4 [9]. In GT 1a, identified NS5A substitutions included M28A/G/T, Q30H/K/R/Y, L31F/M/V, H58D, and Y93H/N/S, while NS3 substitutions included V36L/M, Y56H, V107I, R155I/K, A156G/T/V, V158A, and D168A/G/N/V/Y [9]. Prior to treatment initiation in patients with GT 1a, testing for polymorphisms associated with NS5A resistance, particularly for the resistance-associated variants 28, 30, 31, and 93, is recommended. The addition of ribavirin and extension of treatment duration to 16 weeks overcame baseline NS5A polymorphism resistance. The NS3 resistance-associated variants are less clinically relevant and do not necessarily require testing prior to initiation of therapy. Treatment-emergent NS5A-related substitutions (100% for GTs 1a and 1b) were more persistent in clinical evaluations than NS3-related substitutions (31% for GT 1a and 50% for GT 1b) at week 24 follow-up [9].

### 2.2. Dosing/Pharmacokinetics

Elbasvir/grazoprevir is a fixed dose tablet containing 50 mg of elbasvir and 100 mg of grazoprevir that is given once daily [9, 10]. Elbasvir/grazoprevir is given with or without ribavirin for 12–16 weeks, depending on genotype, treatment experience, and presence of baseline resistance polymorphisms [9]. No dosage adjustments are recommended in patients with renal dysfunction or mild hepatic impairment. Elbasvir/grazoprevir should not be used in patients with moderate to severe hepatic impairment (decompensated cirrhosis) [9].

After administration, peak concentrations are reached at approximately 3 hours and 2 hours for elbasvir and grazoprevir, respectively [9]. Concentrations reach steady-state within 6 days. Elbasvir and grazoprevir are highly protein bound, distributed extensively into most tissues (elbasvir) and predominantly to the liver (grazoprevir). These agents are predominately metabolized by the cytochrome P450 3A4 system, and elimination half-lives for elbasvir and grazoprevir are 24 and 31 hours, respectively, in HCV-infected patients [9].

### 2.3. Adverse Drug Events

In clinical trials, the most frequently reported adverse effects were headache, nausea, fatigue, decreased appetite, anemia, pyrexia, and elevations of ALT [9, 11–15]. Patients treated with elbasvir/grazoprevir should have hepatic function monitored prior to and during treatment.

### 2.4. Drug-Drug Interactions

Elbasvir/grazoprevir has many clinically significant drug-drug interactions [9]. It is contraindicated in patients taking organic anion transporting polypeptides 1B1/3 inhibitors (e.g., cyclosporine) due to significant increases of grazoprevir plasma concentrations when combined. Elbasvir/grazoprevir is also contraindicated with strong cytochrome P450 3A4 inducers (e.g., rifampin, phenytoin, carbamazepine, and St. John's Wort) secondary to decreased concentrations and reduced therapeutic effects of elbasvir and grazoprevir with coadministration [9]. Caution should be used with coadministration of strong cytochrome P450 3A4 inhibitors (e.g., ketoconazole and ritonavir), which may lead to increased concentrations of elbasvir and grazoprevir. Coadministration with nafcillin, bosentan, or modafinil may lead to decreases in elbasvir and grazoprevir concentrations and resultant decreases in therapeutic efficacy, so it is not recommended. Administration with tacrolimus may result in fluctuating plasma tacrolimus concentrations, which should be frequently monitored. Caution should be used with administration of elbasvir/grazoprevir with HMG-CoA reductase inhibitors (atorvastatin, rosuvastatin, fluvastatin, lovastatin, or simvastatin), and clinicians should use the lowest dose HMG-CoA reductase inhibitor possible if this combination is used (max dose: atorvastatin, 20 mg; rosuvastatin, 10 mg) [9]. See HCV-HIV Coinfection section for information about drug-drug interactions with antiretrovirals.

## 3. Genotypes/SVR

Since its identification, HCV has further been subcategorized into 3 different tiers: genotype, subtype, and quasispecies [16]. A total of 6 distinct genotypes have been classified. GTs 1–4 and GT 6 are further categorized, with 67 confirmed subtypes identified [17, 18]. The different genotypes are found in varying geographic regions. GT 1 is the predominate HCV genotype, estimated to comprise 46.2% of all HCV cases worldwide and approximately 70% of cases in the United States [19, 20]. HCV GT 2 is most commonly found in sub-Saharan Africa and Asia and represents approximately 13% of all HCV cases [21]. HCV GT 3 is the second most common genotype, found predominantly in Asia and representing 20–30% of HCV cases [22, 23]. GT 4 affects approximately 12–15% of patients diagnosed worldwide with HCV [23]. This genotype is most prevalent in Northern Africa and the Middle East. HCV GT 5, predominately found in South Africa, is one of the least known genotypes of all HCV strains. As a result, there is minimal treatment data for this subset of patients [24, 25]. GT 6 and its subtypes (previously categorized as GTs 7–11) are predominately found in Asian countries [25, 26]. Excluding subtype 6a, GT 6 remains uncommon [26].

Direct-acting antivirals have become a priority in clinical development to target HCV GT 1 for multiple reasons. Not only is this the most common genotype worldwide, but it also has poor response rates to interferon-based regimens [19, 27]. Fortunately, our DAA armamentarium has dramatically increased since 2011 with the introduction of 9 new agents (12 new individual compounds). These treatment alternatives not only have much higher SVR compared to interferon-based regiments (>90% versus 10–50%) but also have shorter durations of therapy and decreased adverse effects [20]. Specifically, SVR at 12 weeks (SVR12) was observed in 95% of patients receiving immediate treatment with elbasvir/grazoprevir for the treatment of chronic HCV infections [13]. Comparisons of elbasvir/grazoprevir to other newer HCV treatment options are listed in Table 1 [13, 28–32].

## 4. Cost [33]

Prior to 2011, interferon-based regimens were largely utilized. The cost of peginterferon alfa-2a and alfa-2b is approximately $9250 and $8400, respectively, with the addition of a $550–$850 cost of ribavirin for a 12-week course of therapy. However, many patients received prolonged treatment with these regimens due to a lack of observed 2 log_10_ reduction in HCV RNA. As the newer HCV agents became approved, cost has become a significant concern. For a 12-week course of therapy for grazoprevir/elbasvir, the estimated cost is $54,600. While this is substantially higher than the interferon-based treatment, other new alternative therapies are significantly higher (Table 2) [33].

## 5. Clinical Evidence

### 5.1. Treatment-Naïve Patients

The fixed dose combination tablet of elbasvir 50 mg/grazoprevir 100 mg taken orally once daily was approved by the FDA in January 2016. This combination tablet is indicated for a duration of 12 weeks in treatment-naïve patients with or without cirrhosis [9]. The 2016 AASLD/IDSA guidelines list 12 weeks of elbasvir/grazoprevir treatment as a Class 1, Level A recommendation for treatment-naïve patients with GT 1a or 1b, with or without cirrhosis [18]. In GT 1a patients who demonstrate a baseline high-fold change NS5A resistance-associated variants (RAV), treatment duration should be extended to 16 weeks and weight-based ribavirin should be added (AASLD/IDSA Class IIa, Level B recommendation). Elbasvir/grazoprevir treatment for 12 weeks is a Class IIa, Level B recommendation in the AASLD/IDSA guidelines for patients with GT 4 [18].

#### 5.1.1. C-EDGE Treatment-Naïve [13]

The C-EDGE Treatment-Naïve study was a phase III, randomized, blinded, placebo-controlled trial designed to evaluate the safety and efficacy of elbasvir/grazoprevir in patients with chronic HCV GT 1, 4, or 6 [13]. This study included both cirrhotic and noncirrhotic treatment-naïve adults from 60 different centers around the world. Participants were entered into either an immediate or a differed treatment group. Results have only been reported for the immediate treatment group as of publication. Of the participants in the immediate treatment group (*n* = 316), 157 had GT 1a, 131 had GT 1b, 18 had GT 4, and 10 had GT 6. Twenty-two percent (*n* = 70) of participants were classified as cirrhotic. Clinical efficacy was determined by SVR12. Overall, SVR12 was achieved in 95% of participants (299/316); breakdown by GT demonstrated SVR12 of 92% for GT 1a, 99% for GT 1b, 100% for GT 4, and 80% for GT 6. The only virologic breakthrough and a majority of the virologic relapses (9 of 12) occurred in participants with GT 1a. Zeuzem et al. [13] found an association of NS5A RAVs and virologic failure when participants with GT 1a had a shift greater than 5-fold. Safety of elbasvir/grazoprevir was similar to placebo. No serious adverse events were considered to be related to elbasvir/grazoprevir. The most common adverse events were headache (17%) and fatigue (16%). Only 3 participants discontinued the study drug due to adverse events.

#### 5.1.2. C-SCAPE [34]

The C-SCAPE trial was a phase II study designed to evaluate the efficacy and safety of grazoprevir ± elbasvir ± ribavirin in patients with GT 2, 4, 5, or 6. Participants with GT 2 received either the three-drug combination (*n* = 30) or grazoprevir + ribavirin (*n* = 30) [34]. Participants with GT 4, 5, or 6 received either the three-drug combination (*n* = 10 for GT 4, *n* = 4 for GT 5, and *n* = 5 for GT 6) or grazoprevir + elbasvir (*n* = 10 for GT 4, *n* = 4 for GT 5, and *n* = 5 for GT 6). SVR12 for participants with GT 2 was 80% (24/30) for those treated with the three-drug regimen versus 67% (20/30) for those treated with grazoprevir and ribavirin without elbasvir. SVR12 for participants with GT 4 was 100% (10/10) in the three-drug regimen group and 90% (9/10) in the grazoprevir/elbasvir without ribavirin group. For participants with GT 5, the three-drug regimen demonstrated SVR12 of 100% (4/4) while grazoprevir/elbasvir without ribavirin group demonstrated SVR of 25% (1/4). SVR12 for GT 6 participants was 80% (4/5) for both the three-drug regimen group and the grazoprevir/elbasvir without ribavirin group. The authors concluded that patients with GT 4 or 6 should be included into future phase III studies with elbasvir/grazoprevir [34].

#### 5.1.3. C-SWIFT [35]

C-SWIFT was an open-label phase II trial to evaluate treatment duration needed with the combination of elbasvir/grazoprevir + sofosbuvir in treatment-naïve patients with HCV GT 1 or 3 [35]. Both cirrhotic and noncirrhotic patients were included. Noncirrhotic participants with GT 1 received either 4 weeks (*n* = 31) or 6 weeks (*n* = 30) of treatment. Cirrhotic participants with GT 1 received either 6 weeks (*n* = 20) or 8 weeks (*n* = 21) of treatment. Noncirrhotic participants with GT 3 received either 8 weeks (*n* = 15) or 12 weeks (*n* = 14) of treatment while cirrhotic participants received 12 weeks (*n* = 12) of treatment. Interim results have been reported. SVR at 2 weeks for all groups was 100%. SVR at 8 weeks (SVR8) was evaluated for participants with GT 1. Noncirrhotic participants who received 4 weeks of treatment demonstrated SVR8 of 37% (12/31) while those who received 6 weeks of treatment demonstrated SVR8 of 87% (26/30). Cirrhotic patients with GT 1 who received 6 weeks of treatment demonstrated SVR8 of 80% (16/20), while those who received 8 weeks of treatment showed SVR8 of 89% (17/19). Two participants in the 8-week cirrhotic group were excluded for nonvirologic discontinuation. All participants with GT 3 in the noncirrhotic group demonstrated SVR at 4 weeks (SVR4) of 100%, regardless of whether they received 8 weeks or 12 weeks of treatment. Cirrhotic participants with GT 3 demonstrated SVR4 of 90% (9/10). One patient was excluded from this group for nonvirologic discontinuation. The regimen was well tolerated. This study was completed, but final results have yet to be published [36].

#### 5.1.4. C-SALT Part A [37]

C-SALT part A (phase II study) evaluated elbasvir/grazoprevir efficacy and safety in GT 1 patients with Child-Pugh Class B cirrhosis [37]. One aim of this study was to evaluate the pharmacokinetics of elbasvir/grazoprevir in cirrhotic patients. Cirrhotic patients received 50 mg elbasvir + 50 mg grazoprevir daily for 12 weeks. Participants in the noncirrhotic comparator group received the standard elbasvir 50 mg + grazoprevir 100 mg daily for 12 weeks. Interim results showed that all participants with cirrhosis reached undetectable HCV RNA (<15 IU/mL) by the end of treatment and at 4-week follow-up. Participants tolerated the study drug well with no significant hepatotoxicity noted. Cirrhotic patients demonstrated slightly higher plasma grazoprevir levels when compared to their noncirrhotic counterparts. This study has not yet published final results [36].

#### 5.1.5. C-WORTHY [15]

The C-WORTHY trial examined the efficacy and safety of elbasvir/grazoprevir ± ribavirin for GT 1 patients with cirrhosis (treatment-naïve) or treatment-experienced patients with or without cirrhosis [15]. This phase II open-label study randomized treatment-naïve, well-compensated cirrhotic patients (Child-Pugh Class A) to receive elbasvir/grazoprevir (*n* = 29 for 12 weeks, *n* = 31 for 18 weeks) or elbasvir/grazoprevir + ribavirin (*n* = 31 for 12 weeks, *n* = 32 for 18 weeks). In the group of elbasvir/grazoprevir treated for 12 weeks, the SVR12 was 97% (28/29). For participants with ribavirin added and treated for 12 weeks, the SVR12 was 90% (28/31). Participants treated for 18 weeks demonstrated similar results, with SVR12 of 94% (29/31) in the elbasvir/grazoprevir arm and SVR12 of 97% (31/32) in the arm with ribavirin added. All but one of the treatment-naïve patients experiencing virologic failure had GT 1a and NS5A RAVs at virologic failure. As in other studies, the elbasvir/grazoprevir combination was well tolerated.

### 5.2. Treatment-Experienced Patients

The 2016 AASLD/IDSA guidelines list 12 weeks of elbasvir/grazoprevir treatment as a Class 1, Level A recommendation for treatment-experienced patients (prior exposure to peginterferon/ribavirin) with GT 1a or 1b, with or without cirrhosis [18]. In GT 1a patients who demonstrate baseline high-fold change NS5A RAVs, treatment duration should be extended to 16 weeks and weight-based ribavirin should be added (AASLD/IDSA Class I, Level B recommendation). For GT 1 patients who previously failed a NS3 protease inhibitor, elbasvir/grazoprevir plus weight-based ribavirin for 12-week duration (16 weeks if high-fold change NS5A RAV in GT 1a) is a Class IIa, Level B recommendation for patients with or without compensated cirrhosis. Elbasvir/grazoprevir treatment for 12 weeks is a Class IIa, Level B recommendation in the AASLD/IDSA guidelines for patients with GT 4 (cirrhotic and noncirrhotic) who experienced virologic relapse after treatment with peginterferon/ribavirin. Weight-based ribavirin should be added to the regimen for GT 4 patients who experienced virologic failure while on peginterferon/ribavirin, and treatment duration should be extended to 16 weeks (Class IIa, Level B) [18].

#### 5.2.1. C-WORTHY [15]

The C-WORTHY trial examined the efficacy and safety of elbasvir/grazoprevir ± ribavirin for treatment-experienced GT 1 patients (null responders) with or without cirrhosis [15]. Treatment-naïve cirrhotic patients were also included in this study, with those results reported in the previous section. This phase II open-label study randomized patients who had previously failed peginterferon + ribavirin to receive elbasvir/grazoprevir (*n* = 33 for 12 weeks, *n* = 32 for 18 weeks) or elbasvir/grazoprevir + ribavirin (*n* = 32 for 12 weeks, *n* = 33 for 18 weeks). Cirrhotic patients represented 34–42% of the participants in the four groups. In the elbasvir/grazoprevir 12-week treatment group, the SVR12 was 91% (30/33). For participants who received elbasvir/grazoprevir + ribavirin for 12 weeks, the SVR12 was 94% (30/32). Participants treated for 18 weeks demonstrated similar results, with SVR12 of 97% (31/32) in the elbasvir/grazoprevir arm and SVR12 of 100% (33/33) in the arm with ribavirin added. All four treatment failures demonstrated NS5A RAVs at the time of virologic failure.

#### 5.2.2. C-EDGE Treatment-Experienced [38]

The C-EDGE Treatment-Experienced phase III trial evaluated the safety and efficacy of elbasvir/grazoprevir ± ribavirin for GT 1 or GT 4 patients who previously failed peginterferon + ribavirin [38]. Both cirrhotic and noncirrhotic patients were included. Prior treatment failure included null responders and partial responders/relapses. Participants received either elbasvir/grazoprevir (*n* = 105) or elbasvir/grazoprevir + ribavirin (*n* = 104) for 12 weeks. Overall, SVR at 4 weeks (SVR4) was 95% (100/105) for the elbasvir/grazoprevir group and 98% (102/104) for the group with ribavirin added. In subgroup analysis, 95% (55/58) with GT 1a in the elbasvir/grazoprevir group achieved SVR4 while 96% (54/56) in the ribavirin added group achieved SVR4. All participants with GT 1b (*n* = 38 in elbasvir/grazoprevir, *n* = 33 in added ribavirin) achieved SVR4. Fewer participants had GT 4; 78% (7/9) in the elbasvir/grazoprevir group achieved SVR4, while 100% (15/15) in the added ribavirin group reached SVR4. Of note, one of the treatment failures was a participant who died of lymphoma during the study. Both null responders (and partial/relapse participants) responded well to elbasvir/grazoprevir (SVR4 93% [42/45] and 98% [59/60], resp.). When ribavirin was added to the regimen, null responders reached an SVR4 of 95% (42/44) and partial/relapse participants achieved an SVR4 of 100% (60/60). Participants coinfected with HIV were included in this study; all coinfected participants reached SVR4 (6/6 in elbasvir/grazoprevir, 5/5 with added ribavirin). Cirrhotic participants responded well, with SVR4 92% (34/37) in the elbasvir/grazoprevir group and SVR4 97% (35/36) in the ribavirin added group. Full study results have yet to be reported.

#### 5.2.3. C-SALVAGE [39, 40]

The C-SALVAGE phase II study examined the use of elbasvir/grazoprevir + ribavirin for participants with GT 1 who had previously failed combination therapy of peginterferon + ribavirin + HCV NS3/4A protease inhibitor [39, 40]. Both cirrhotic and noncirrhotic participants were included. NS3 RAVs were detected in 34 participants at baseline (30 with ≤5-fold change, 4 with >5-fold change). NS5A RAVs were detected in 8 patients at baseline (3 with ≤5-fold change, 5 with >5-fold change). All participants (*n* = 79) received elbasvir 50 mg/grazoprevir 100 mg daily + weight-based ribavirin. Overall, SVR12 was 96% (76/79). This rate was maintained for an additional 12 weeks and demonstrated comparable SVR results at 24 weeks. All three virologic failures were relapses. Each of these participants had NS3 RAVs at baseline (2 with ≤5-fold change, 1 with >5-fold change). Two of the failures also had NS5A RAVs at baseline, both with >5-fold change. Both of the failures with NS5A RAVs were also cirrhotic (1 GT 1a, 1 GT 1b), while the other failure was noncirrhotic GT 1a. The authors concluded that elbasvir/grazoprevir + ribavirin for 12 weeks is a viable therapeutic option for patients who had previously failed therapy, including those with NS3 variants [39, 40].

### 5.3. HCV-HIV Coinfection

There are over 4 million people worldwide who are coinfected with HIV and HCV, which equates to an overall prevalence of 6.2% of all HIV-infected individuals [41]. HIV infection has demonstrated increased progression of HCV-associated liver dysfunction and has been associated with higher rates of all-cause, liver-related, and AIDS-related death [42]. Coinfected patients receiving HIV-antiretroviral therapy (ART) tend to have delayed progression to cirrhosis, especially if the HIV viral load is undetectable [43]. Concurrent treatment of HCV in these patients is common. The AASLD/IDSA guidelines recommend the use of daily elbasvir/grazoprevir in patients with HIV/HCV coinfection when the patient is not taking ART with clinically significant drug interactions (Class IIa, Level B) [18]. Elbasvir/grazoprevir was tested in coinfected patients in two trials: C-WORTHY and C-EDGE coinfection.

#### 5.3.1. C-WORTHY Coinfection [44]

Elbasvir/grazoprevir was studied in 59 patients with HIV and HCV coinfection without cirrhosis as a substudy of the larger C-WORTHY phase 2 trial [44]. Patients with GT 1, an undetectable HIV viral load for >24 weeks, and a CD4 T-cell count >300 cells/mm^3^ at screening were included. The allowed HIV ART consisted of at least 8 weeks with tenofovir or abacavir plus emtricitabine or lamivudine plus raltegravir. Patients received elbasvir/grazoprevir ± ribavirin for 12 weeks. There were 46 patients with GT 1a, of which 24 (52%) patients received ribavirin. Of the 13 patients with GT 1b, only 5 (38%) patients received ribavirin. Of the 29 patients who received ribavirin, the SVR12 rate was 97% (28/29 patients), while the SVR12 of the 30 patients who did not receive ribavirin was 87% (26/30 patients). The regimens were generally well-tolerated, with the most common adverse effects being mild-to-moderate fatigue, headache, nausea, and diarrhea.

#### 5.3.2. C-EDGE Coinfection [12]

In this prospective single-arm clinical trial, elbasvir/grazoprevir was studied in 218 patients with treatment-naïve chronic HCV (GT 1, 4, or 6) and HIV coinfection, both with and without cirrhosis [12]. Participants could either be naïve to ART or be on stable ART with tenofovir or abacavir plus emtricitabine or lamivudine plus either raltegravir, dolutegravir, or rilpivirine for at least 8 weeks. ART-naïve patients were required to have an HIV viral load <50,000 copies/mL and a CD4 T-cell count >500 cells/*μ*L, while patients who were stable on ART were required to have an undetectable HIV viral load. Participants received elbasvir 50 mg/grazoprevir 100 mg once daily for 12 weeks. Ninety-seven percent (211/218) of participants were on antiretroviral therapy with an undetectable HIV viral load with a median CD4 T-cell count of 568 cells/*μ*L. Sixteen percent (35/218) of participants had compensated cirrhosis. Overall, 210/218 (96.3%) patients achieved SVR12: 139/144 (96.5%) with GT 1a, 42/44 (95.5%) with GT 1b, and 27/28 (96.4%) with GT 4. All cirrhotic patients achieved SVR12. The regimen was generally well tolerated with the most common adverse effects being fatigue (13%), headache (12%), and nausea (9%).

#### 5.3.3. Drug-Drug Interactions [45]

HCV-HIV coinfected patients present a particular problem when selecting not only HCV regimens but also HIV regimens. Elbasvir/grazoprevir was studied in HIV patients whose ART regimens did not contain an HIV-protease inhibitor. In particular, atazanavir, darunavir, lopinavir, saquinavir, and tipranavir are protease inhibitors whose use is contraindicated with elbasvir/grazoprevir because elevated concentrations of elbasvir/grazoprevir have been observed, leading to elevated alanine aminotransferase (ALT) levels [45]. Additionally, patients in the two trials presented above were excluded if taking efavirenz, as this nonnucleoside reverse transcriptase inhibitor is contraindicated due to reductions in the concentrations of elbasvir/grazoprevir by as much as 80% [45]. Two other ART agents, cobicistat and ritonavir, although not contraindicated, should be used with caution if coadministered with elbasvir/grazoprevir [45].

### 5.4. Patients with Stage 4 or 5 Chronic Kidney Disease

The AASLD/IDSA guidelines recommend 12 weeks of daily elbasvir 50 mg/grazoprevir 100 mg for treatment of patients with HCV GT 1a, 1b, or 4 and severe renal impairment (creatinine clearance of <30 mL/min) (Class IIb, Level B) [18].

#### 5.4.1. C-SURFER [11]

The phase III C-SURFER study focused on patients with HCV GT 1 with stage 4 or 5 chronic kidney disease (CKD), including patients receiving hemodialysis [11]. Safety and efficacy of elbasvir 50 mg/grazoprevir 100 mg daily for 12 weeks were evaluated. Participants were randomized to either an immediate treatment group (*n* = 111) or a placebo (delayed treatment) group (*n* = 113); eleven additional participants were included in a pharmacokinetic arm, receiving immediate treatment (*n* = 235 total participants). Fifty-two percent of participants had GT 1a. Seventy-six percent of participants (179/235) were on hemodialysis, 6% (14/235) had cirrhosis, and 80% (189/235) were treatment-naïve. In the immediate treatment + pharmacokinetic group, SVR12 was achieved in 94% (115/122) of participants. In a modified analysis, 6 patients were excluded for reasons other than virologic failure, resulting in a SVR12 of 99% (115/116). Adverse effects were similar between immediate treatment and placebo, with most adverse effects classified as mild to moderate in both groups. The authors concluded that elbasvir/grazoprevir for 12 weeks is an effective treatment option for GT 1 patients with stage 4 or 5 CKD [11].

### 5.5. Patients Who Inject IV Drugs Receiving Opioid Agonist Therapy

Injection drug use is a major risk factor for HCV infection, accounting for 50–80% of all HCV infections [46]. Study results from the trial presented below are not yet incorporated into the AASLD/IDSA guidelines but show promise for this significant population.

#### 5.5.1. C-EDGE CO-STAR [47]

The C-EDGE CO-STAR trial evaluated the efficacy of elbasvir/grazoprevir treatment for 12 weeks in patients who inject intravenous drugs and were currently receiving opioid agonist therapy [47]. Participants with GT 1, 4, or 6 were included, with or without HIV coinfection ± cirrhosis. A total of 301 patients were randomized in 2 : 1 ratio to receive immediate or delayed treatment. Ninety-nine percent (199/201) of participants in the immediate treatment group completed 12-week treatment, with SVR4 96% (193/201). Four of these participants experienced virologic failure, detected after completion of therapy. Adverse effects were similar between the two groups. The authors concluded that preliminary data demonstrate that elbasvir/grazoprevir is safe and effective in the special population of intravenous drug users receiving opioid agonist therapy [47]. This study is currently ongoing [36] (ClinicalTrials.gov).

## 6. Conclusion

Elbasvir/grazoprevir has earned a spot in the current hepatitis C guidelines as a first-line option for treatment-naïve patients with GT 1a or 1b, with or without cirrhosis. It is also recommended for treatment-experienced patients with GT 1a or 1b. For patients with GT 4, elbasvir/grazoprevir is recommended for both treatment-naïve and treatment-experienced patients, with or without cirrhosis [18]. Elbasvir/grazoprevir may be used in patients with HIV coinfection or severe renal impairment. Although it does have more drug-drug interactions than other treatments, elbasvir/grazoprevir is the most cost-effective. Given the efficacy and safety data described above, elbasvir/grazoprevir should be considered a potent new weapon in the fight against chronic hepatitis C.

## Figures and Tables

**Table 1:** SVR_12_ rates of first-line therapies for the treatment of HCV genotypes 1a, 1b, and 4 [13, 28–32].

	SVR_12_ in treatment-naïve patients			
	Overall	Genotype 1a	Genotype 1b	Genotype 4
Grazoprevir-elbasvir [13]	95%	92%	99%	100%
Ledipasvir-sofosbuvir [28]	100%	NA	NA	NA
Paritaprevir-ritonavir-ombitasvir + dasabuvir [29]	91.8%	92.2%	100%	NA
Simeprevir + sofosbuvir [30, 31]	93%	NA	NA	NA
Daclatasvir + sofosbuvir [32]	100%	NA	NA	NA

NA: not applicable.

**Table 2:** Cost of guideline recommended therapies for HCV genotype 1a [33].

	Elbasvir-grazoprevir (Zepatier)	Simeprevir (Olysio) + sofosbuvir (Sovaldi)	Ledipasvir-sofosbuvir (Harvoni)	Ombitasvir-paritaprevir-ritonavir-dasabuvir (Viekira) + ribavirin	Daclatasvir (Daklinza) + sofosbuvir (Sovaldi)
8 weeks	NA	NA	$63,000	NA	NA
12 weeks	$54,600	$150,000	$94,500	$84,019	$147,000
16 weeks	$72,800	NA	NA	NA	NA
24 weeks	NA	NA	$189,000	$168,038	NA

NA: not applicable.
